# Supplementary material for: SARS-CoV-2 N-Antigen Quantification in Respiratory Tract, Plasma and Urine: Kinetics and Association with RT-qPCR Results
Source: Viruses. 2023 Apr 24;15(5):1041. doi: 10.3390/v15051041 (PMC10221862; doi:10.3390/v15051041)
Supplement: Supplementary file 1 [file viruses-15-01041-s001.zip › viruses-2343857-supplementary.pdf]

Supplementary Table S1: Clinical and biological features of all patients

	N	Median [Minimum,Maximum]; Or n (%)
**Time between first positive test and inclusion (days)**	26	3 [0 ; 11]
**Age (years)**	26	67 [27 ; 86]
**Comorbidities**		
Chronic cardiac disease	25	7 (28 %)
Hypertension	23	8 (35 %)
Chronic pulmonary disease	25	5 (20 %)
Asthma	25	2 (8.0 %)
Chronic kidney disease	25	1 (4.0 %)
Obesity	26	8 (31 %)
Diabetes	24	3 (12 %)
**ICU admission**	26	25 (96 %)
**Death while in hospital**	26	12 (46 %)
**Symptoms**		
Fever	26	23 (88 %)
Cough	26	16 (62 %)
Diarrhea	26	6 (23 %)
Anosmia	14	2 (14 %)
**Biology**		
Hemoglobin (g/dL)	22	14.8 [10.0 ; 17.1]
Lymphocyte (G/L)	21	0.70 [0.28 ; 2.76]
Platelets (G/L)	21	178 [121 ; 337]
ALT (U/L)	16	46.0 [1.3 ; 660.0]
AST (U/L)	16	72.0 [29.1 ; 596.0]
Total Bilirubin (μmol/L)	14	12.0 [4.0 ; 37.0]
Urea (mmol/L)	21	7.0 [3.0 ; 15.1]
Creatinin (μmol/L)	22	81 [70 ; 209]
C-reactive protein (mg/L)	17	136 [10 ; 223]

Supplementary Table S2: Members of French COVID cohort study group.

Prénom	*Nom*	*@*	*Affiliation*
Laurent	ABEL	laurent.abel@inserm.fr	Inserm UMR 1163, Paris, France
Amal	ABROUS	amal.abrous@inserm.fr	Inserm sponsor, Paris, France
Claire	ANDREJAK	andrejak.claire@chu-amiens.fr	CHU Amiens, France
François	ANGOULVANT	francois.angoulvant@aphp.fr	Hôpital Necker, Paris, France
Delphine	BACHELET	delphine.bachelet@aphp.fr	Hôpital Bichat, Paris, France
Marie	BARTOLI	marie.bartoli@anrs.fr	ANRS, Paris, France
Sylvie	BEHILILL	sylvie.behillil@pasteur.fr	Pasteur Institute, Paris, France
Marine	BELUZE	marine.beluze@aphp.fr	F-CRIN Partners Platform, Paris, France
Krishna	BHAVSAR	krishna.bhavsar@aphp.fr	Hôpital Bichat, Paris, France
Lila	BOUADMA	lila.bouadma@aphp.fr	Hôpital Bichat, Paris, France
Minerva	CERVANTES-GONZALEZ	minerva.cervantes@inserm.fr	Hôpital Bichat, Paris, France
Anissa	CHAIR	anissa.chair@aphp.fr	Hôpital Bichat, Paris, France
Charlotte	CHARPENTIER	charlotte.charpentier@aphp.fr	Hôpital Bichat, Paris, France
Léo	CHENARD	leo.chenard@aphp.fr	Hôpital Bichat, Paris, France
Catherine	CHIROUZE	catherine.chirouze@univ-fcomte.fr	CHRU Jean Minjoz, Besançon, France
Sandrine	COUFFIN-CADIERGUES	sandrine.couffin-cadiergues@inserm.fr	Inserm sponsor, Paris, France
Camille	COUFFIGNAL	camille.couffignal@aphp.fr	Hôpital Bichat, Paris, France
Nathalie	DE CASTRO	nathalie.de-castro@aphp.fr	Hôpital Saint Louis, Paris, France
Marie-Pierre	DEBRAY	marie-pierre.debray@aphp.fr	Hôpital Bichat, Paris, France
Dominique	DEPLANQUE	Dominique.DEPLANQUE@chru-lille.fr	Hôpital Calmette, Lille, France
Diane	DESCAMPS	diane.descamps@aphp.fr	Hôpital Bichat, Paris, France
Alpha	DIALLO	alpha.diallo@inserm.fr	ANRS, Paris, France
Fernanda	DIAS DA SILVA	fernanda.dias-da-silva@inserm.fr	Inserm sponsor, Paris, France

Céline	DORIVAL	celine.dorival@iplesp.upmc.fr	Inserm UMR 1136, Paris, France
Xavier	DUVAL	xavier.duval@aphp.fr	Hôpital Bichat, Paris, France
Philippine	ELOY	philippine.eloy@aphp.fr	Hôpital Bichat, Paris, France
Vincent	ENOUF	vincent.enouf@pasteur.fr	Pasteur Institute, Paris, France
Hélène	ESPEROU	helene.esperou@inserm.fr	Inserm sponsor, Paris, France
Marina	ESPOSITO-FARESE	marina.esposito-farese@aphp.fr	Hôpital Bichat, Paris, France
Manuel	ETIENNE	Manuel.Etienne@chu-rouen.fr	CHU Rouen, France
Aline-Marie	FLORENCE	aline-marie.florence@aphp.fr	Hôpital Bichat, Paris, France
Alexandre	GAYMARD	alexandre.gaymard@chu-lyon.fr	Inserm UMR 1111, Lyon, France
Jade	GHOSN	jade.ghosn@aphp.fr	Hôpital Bichat, Paris, France
Tristan	GIGANTE	T.GIGANTE@chru-nancy.fr	F-CRIN INI-CRCT, Nancy, France
Morgane	GILG	M.GILG@chru-nancy.fr	F-CRIN INI-CRCT, Nancy, France
François	GOEHRINGER	f.goehringer@chru-nancy.fr	CHU Nancy, France
Jérémie	GUEDJ	jeremie.guedj@inserm.fr	Inserm UMR 1137, Paris, France
Ikram	HOUAS	ikram.houas@inserm.fr	Inserm sponsor, Paris, France
Isabelle	HOFFMANN	isabelle.hoffmann@aphp.fr	Hôpital Bichat, Paris, France
Jean-Sébastien	HULOT	jean-sebastien.hulot@aphp.fr	Hôpital Européen Georges Pompidou, Paris, France
Salma	JAAFOURA	salma.jaafoura@inserm.fr	Inserm sponsor, Paris, France
Simon	JAMARD	simon.jamard@univ-tours.fr	Hôpital Bretonneau, Tours, France
Ouifiya	KAFIF	ouifiya.kafif@aphp.fr	Hôpital Bichat, Paris, France
Antoine	KHALIL	antoine.khalil@aphp.fr	Hôpital Bichat, Paris, France
Nadhem	LAFHEJ	nadhem.lafhej@aphp.fr	Hôpital Bichat, Paris, France
Cédric	LAOUÉNAN	cedric.laouenan@aphp.fr	Hôpital Bichat, Paris, France
Samira	LARIBI	samira.laribi@aphp.fr	Hôpital Bichat, Paris, France
Minh	LE	minh.le@aphp.fr	Hôpital Bichat, Paris, France
Quentin	LE HINGRAT	quentin.lehingrat@aphp.fr	Hôpital Bichat, Paris, France
Soizic	LE MESTRE	soizic.le mestre@anrs.fr	ANRS-MIE, Paris, France

Sophie	LETROU	sophie.letrou@aphp.fr	Hôpital Bichat, Paris, France
Yves	LEVY	yves.levy@inserm.fr	Vaccine Research Insitute (VRI), Inserm UMR 955, Créteil, France
Bruno	LINA	bruno.lina@chu-lyon.fr	Inserm UMR 1111, Lyon, France
Guillaume	LINGAS	guillaume.lingas@inserm.fr	Inserm UMR 1137, Paris, France
Denis	MALVY	denis.malvy@chu-bordeaux.fr	CHU Bordeaux, France
France	MENTRÉ	france.mentre@inserm.fr	Hôpital Bichat, Paris, France
Hugo	MOUQUET	hugo.mouquet@pasteur.fr	Pasteur Institute, Paris, France
Nadège	NEANT	nadege.neant@inserm.fr	Inserm UMR 1137, Paris, France
Christelle	PAUL	christelle.paul@anrs.fr	ANRS-MIE, Paris, France
Aurélie	PAPADOPOULOS	aurelie.papadopoulos@inserm.fr	Inserm sponsor, Paris, France
Ventzislava	PETROV-SANCHEZ	ventzislava.petrov-sanchez@anrs.fr	ANRS-MIE, Paris, France
Gilles	PEYTAVIN	gilles.peytavin@aphp.fr	Hôpital Bichat, Paris, France
Valentine	PIQUARD	valentine.piquard@aphp.fr	Hôpital Bichat, Paris, France
Olivier	PICONE	olivier.picone@aphp.fr	Hôpital Louis Mourier, Colombes, France
Manuel	ROSA-CALATRAVA	manuel.rosa-calatrava@univ-lyon1.fr	Inserm UMR 1111, Lyon, France
Bénédicte	ROSSIGNOL	B.ROSSIGNOL@chru-nancy.fr	F-CRIN INI-CRCT, Nancy, France
Patrick	ROSSIGNOL	p.rossignol@chru-nancy.fr	CHU Nancy, France
Carine	ROY	carine.roy@aphp.fr	Hôpital Bichat, Paris, France
Marion	SCHNEIDER	marion.schneider2@aphp.fr	Hôpital Bichat, Paris, France
Coralie	TARDIVON	coralie.tardivon@aphp.fr	Hôpital Bichat, Paris, France
Jean-François	TIMSIT	jean-francois.timsit@aphp.fr	Hôpital Bichat, Paris, France
Sarah	TUBIANA	sarah.tubiana@aphp.fr	Hôpital Bichat, Paris, France
Sylvie	VAN DER WERF	sylvie.van-der-werf@pasteur.fr	Pasteur Institute, Paris, France
Benoit	VISSEAUX	benoit.visseaux@aphp.fr	Hôpital Bichat, Paris, France
Aurélie	WIEDEMANN	aurelie.wiedemann@inserm.fr	Vaccine Research Insitute (VRI), Inserm UMR 955, Créteil, France
